# Supplementary material for: Durable, Ultrathin, and Antifouling Polymer Brush Coating for Efficient Condensation Heat Transfer
Source: ACS Appl Mater Interfaces. 2023 Dec 20;16(1):1941–9. doi: 10.1021/acsami.3c17293 (PMC10788830; doi:10.1021/acsami.3c17293)
Supplement: Supplementary file 1 — am3c17293_si_001.pdf [file am3c17293_si_001.pdf]

Supporting Information for

Durable, ultrathin, and antifouling polymer brush coating for efficient condensation heat transfer

Shuai Li[1], Cheuk Wing Edmond Lam[2#], Matteo Donati[2], Kartik Regulagadda[2], Emre Yavuz[1], Till Pfeiffer[3], Panagiotis Sarkiris[4], Evangelos Gogolides[4], Athanasios Milionis[2], Dimos Poulikakos[2], Hans-Jürgen Butt[1], Michael Kappl[1*]*

1. Max Planck Institute for Polymer Research, 55128 Mainz, Germany.

2. Laboratory of Thermodynamics in Emerging Technologies, Department of Mechanical and Process Engineering, ETH Zurich, 8092 Zurich, Switzerland.

3. Institute for Technical Thermodynamics, Technical University of Darmstadt, 64287 Darmstadt, Germany.

4. Institute of Nanoscience and Nanotechnology, NCSR "Demokritos", 15341, Agia Paraskevi, Attiki, Greece

E-mail: dpoulikakos@ethz.ch; kappl@mpip-mainz.mpg.de

Keywords: dropwise condensation, heat transfer, transition, wetting, polydimethylsiloxane, durability

This file includes:

Figures S1 to S11

Tables S1 to S3

Videos S1 to S11

Supporting References

Supplementary Figures

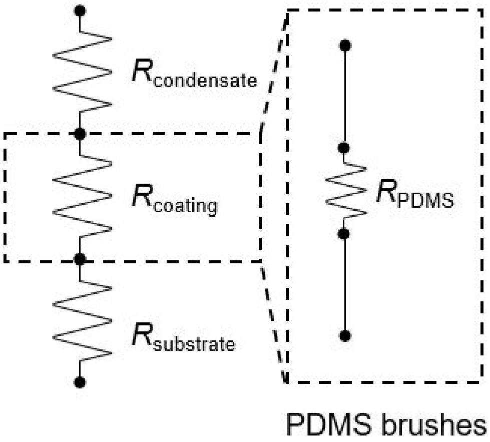

Figure S1. Thermal resistance. Schematic of the thermal resistance network of PDMS brushes.

Based on the assumption of one-dimensional heat conduction, thermal resistance (R) of three surfaces are calculated.[1-3] For polydimethylsiloxane (PDMS) brushes, thermal resistance is calculated simply using $R_{PDMS} = d_{PDMS}/k_{PDMS}$, where d_{PDMS} and k_{PDMS} is coating thickness and thermal conductivity, respectively. Thermal conductivity PDMS is set to 0.16 $W \cdot m^{-1} \cdot K^{-1}$.

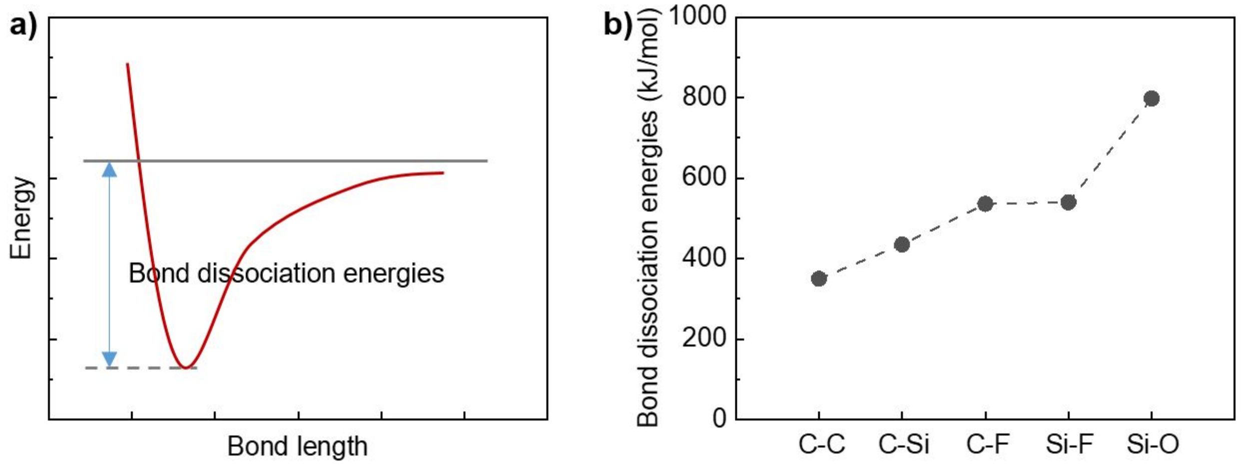

Figure S2. Bond dissociation energies. a) Schematic showing the definition of bond dissociation energies: the energy required to break a bond and form two atomic or molecular fragments.[4] b) Comparison of bond dissociation energies for different bonds.[5]

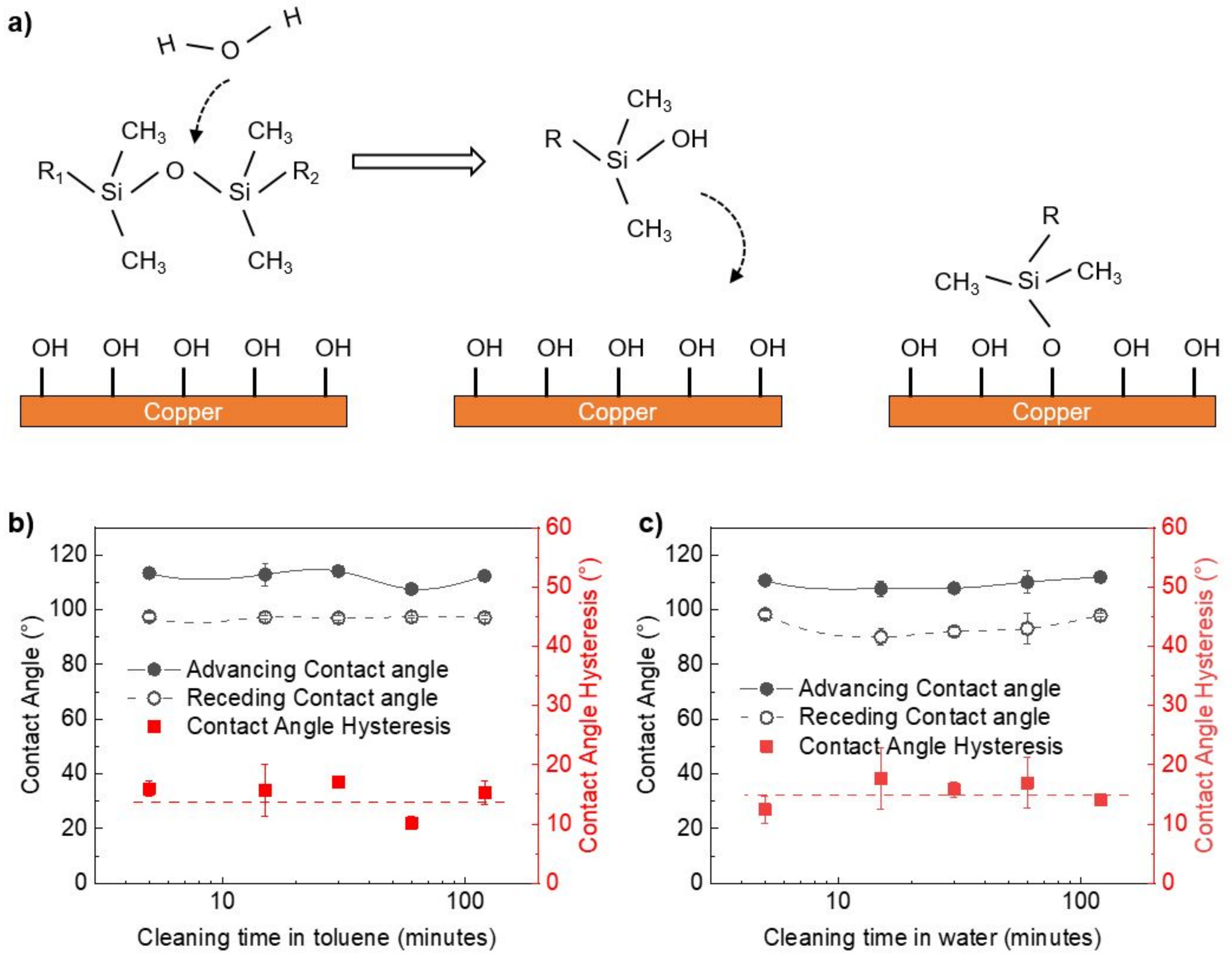

Figure S3. a) Schematic showing the bonding process of PDMS brushes on the copper. b) Water advancing contact angle, receding contact angle, and contact angle hysteresis on PDMS brushes after ultrasonic cleaning in toluene for different times. c) Water advancing contact angle, receding contact angle, and contact angle hysteresis on PDMS brushes after ultrasonic cleaning in water for varying time.

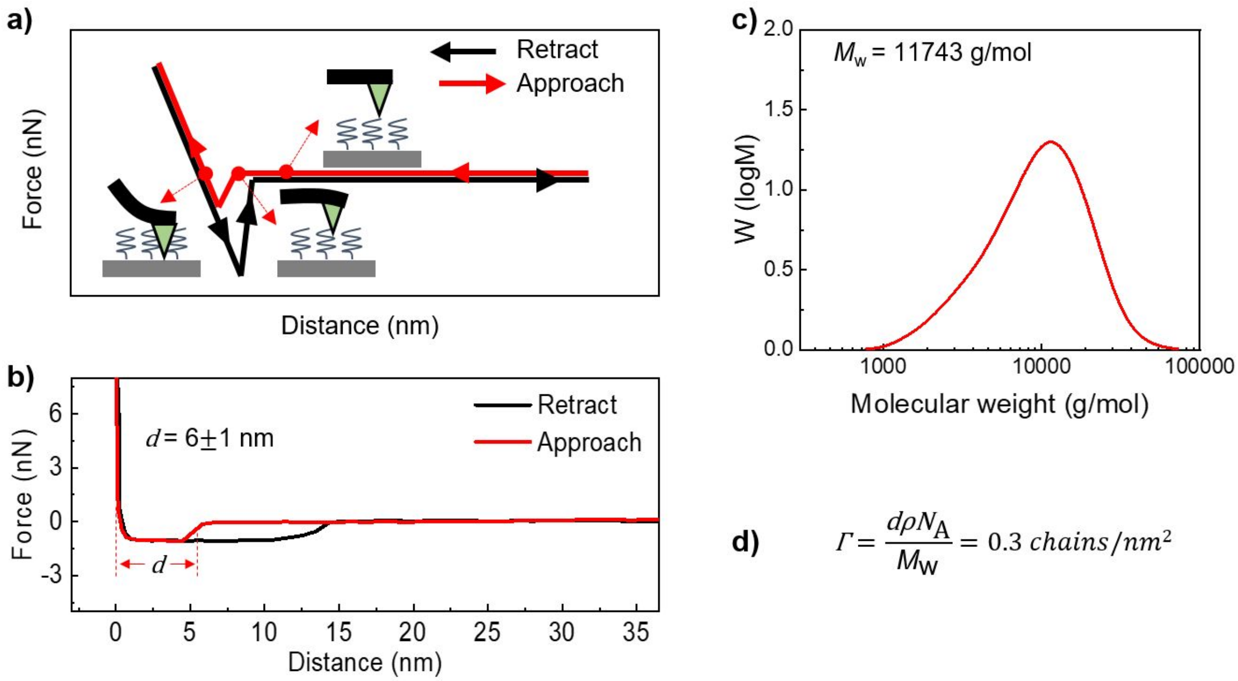

Figure S4. Estimation of grafting density of PDMS brushes. a) Schematic showing the state of the AFM cantilever and tip corresponding to different positions during the approach and retract phases during a force curve. b) Representative force curves measured by AFM. The brush thickness is extracted as the distance between onset of attractive force (AFM tip touching the brush surface) and the hard wall repulsion (tip penetrating the brush). The measured brush thickness d is 6 nm ± 1 nm. c) Molecular weight distribution and average molecular weight M_w measured by gel permeation chromatography. d) Equation used for calculation of grafting density Γ, where ρ and N_A represent mass density and Avogadro constant, respectively.

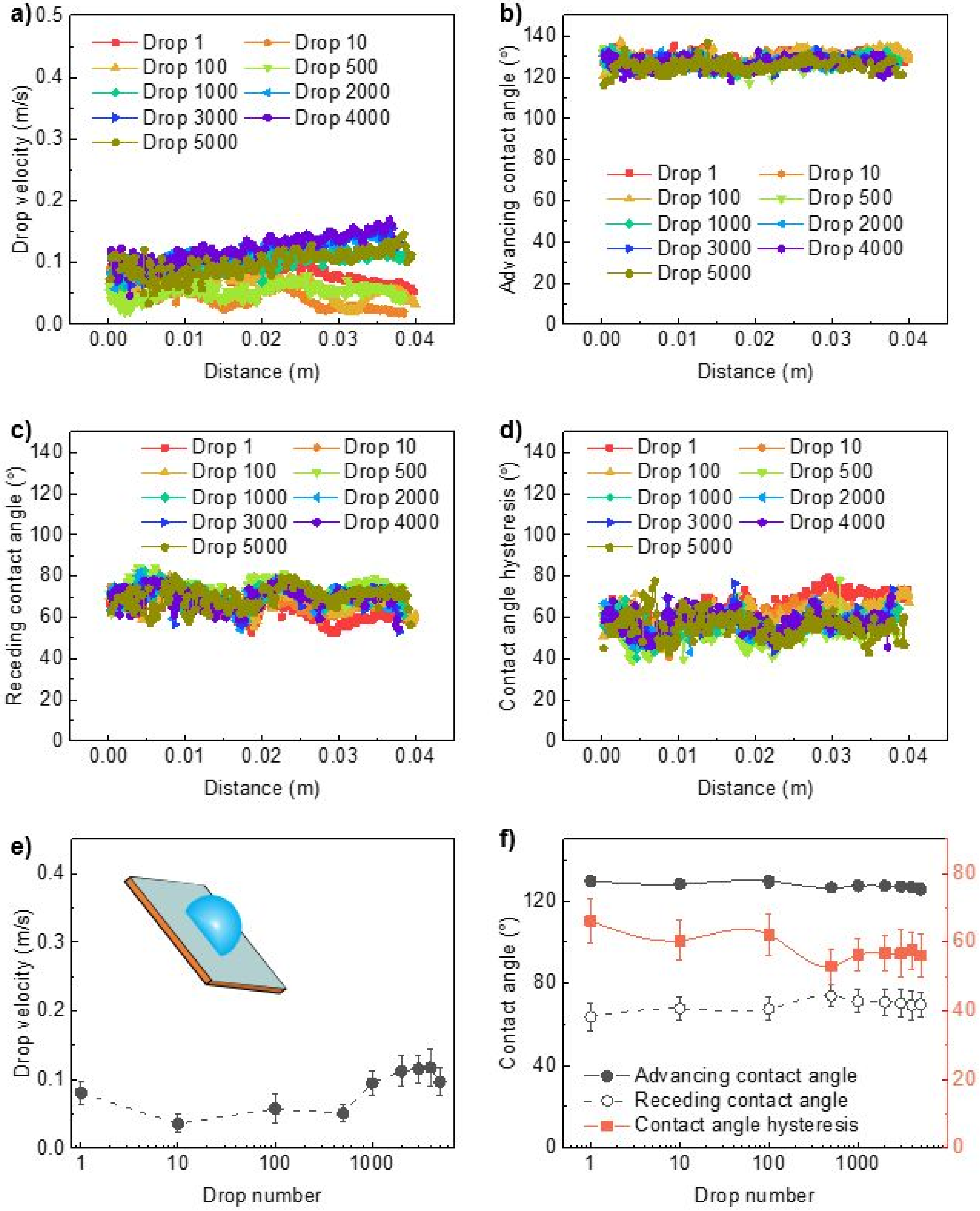
a)
b)
c)
d)
e)
f)
Drop 1
Drop 10
Drop 100
Drop 500
Drop 1000
Drop 2000
Drop 3000
Drop 4000
Drop 5000
Drop velocity (m/s)
Advancing contact angle (°)
Receding contact angle (°)
Contact angle hysteresis (°)
Distance (m)
Drop number
Contact angle (°)
Advancing contact angle
Receding contact angle
Contact angle hysteresis

Figure S5. Continuous water drop sliding on PDMS brushes coated copper plate. a) Velocity of successive water drops as a function of displacement. b) Advancing contact angle of successive water drops as a function of displacement. c) Receding contact angle of successive water drops as a function of displacement. d) Contact angle hysteresis of successive water drops as a function of displacement. Surface tilt angle: 50°. Drop volume: 45 μL. e) Sliding velocity of successive droplets on tilted PDMS brushes. Surface tilt angle: 50°. Drop volume: 45 μL. Inset: Schematic of water drop slide on the surface. f) Water advancing and receding contact angles, and contact angle hysteresis of successive droplets on tilted PDMS brushes.

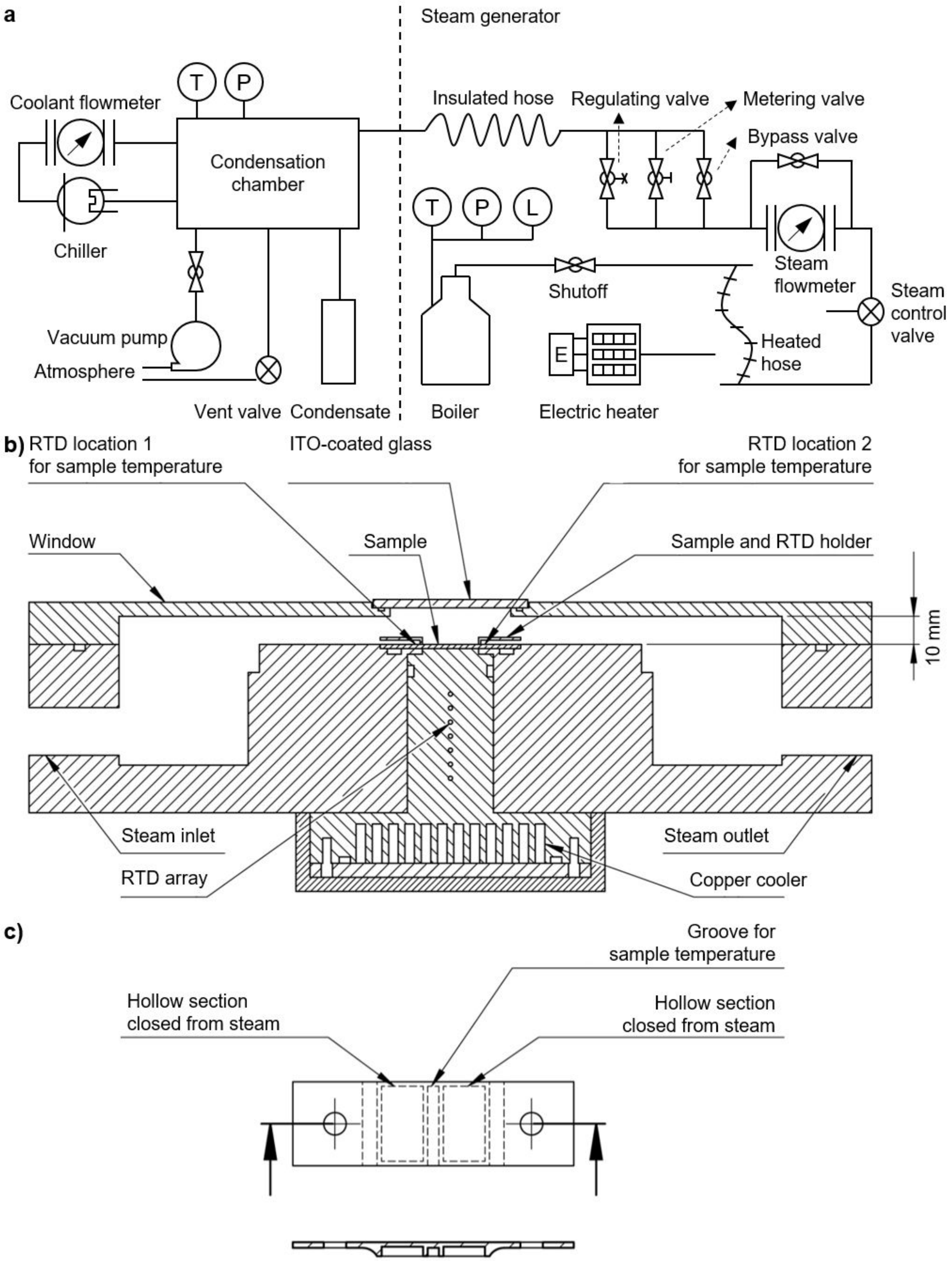
a
Steam generator
Coolant flowmeter
T
P
Condensation chamber
Chiller
Vacuum pump
Atmosphere
Vent valve
Condensate
Insulated hose
Regulating valve
Metering valve
Bypass valve
T
P
L
Shutoff
Steam flowmeter
Steam control valve
Heated hose
E
Boiler
Electric heater
b)
RTD location 1 for sample temperature
ITO-coated glass
RTD location 2 for sample temperature
Window
Sample
Sample and RTD holder
10 mm
Steam inlet
Steam outlet
RTD array
Copper cooler
c)
Groove for sample temperature
Hollow section closed from steam
Hollow section closed from steam

Figure S6. Device for condensation heat transfer measurements at 30 mbar. a) Schematic of the overall experimental setup. b) Top-view cross-section of the condensation chamber. c) Schematic of sample and surface temperature RTD holder, top view and cross-section.

For low-pressure heat transfer measurement, we follow similar procedures to our previous work, which is referred to for details.[1] A short summary is provided below. In this current work, low-pressure heat transfer measurement is conducted at 30 mbar.

To minimize non-condensable gases in the testing chamber, deionized water is continuously boiled at > 1.4 bar, draining the steam for 30 minutes and the condensation chamber is vacuumed to < 0.01 mbar (1 Pa) at the same time. During experiments, steam is generated at 1.4 ± 0.01 bar and its flow rate is measured by a flowmeter (FAM3255, ABB). Regulating and metering valves reduce the pressure and temperature of the steam as it enters the chamber to 30 ± 0.5 mbar and ~24 ˚C (corresponding saturation temperature). The steam is at saturation. A vacuum pump (RC 6, VACUUBRAND) maintains a continuous flow of the low-pressure saturated steam horizontally across the surface. The resulting mean steam speed in the channel is ~ 4.6 $m \cdot s^{-1}$. The copper cooler at the back of the test surface is cooled by a recirculating chiller (WKL 2200, LAUDA), where the flow rate is monitored by a flowmeter (SITRANS FM MAG5000 and SITRANS FM MAG 1100, SIEMENS). An array of 7 RTDs is in the cooler to obtain the heat flux through it with a linear fit. In the chamber, the surface temperature is monitored continuously with two RTDs. The chamber steam temperature is similarly monitored with two RTDs. A capacitance gauge is used to monitor the chamber steam pressure. To avoid condensation before chamber steam conditions are stabilized at the said pressure and temperatures, the chiller is set to an initial coolant temperature of 25 °C, higher than the target steam temperature. Once steam is stabilized, the coolant temperature is reduced to trigger condensation at 7 set points. As the system reaches steady state at each chiller

set point, the coolant flow rate is set to 180 ± 10 $L \cdot h^{-1}$ and measurements are taken over 1 minute, during which the chamber pressure has to maintain at 30 ± 0.5 mbar and the boiler pressure at 1.4 ± 0.01 bar without intervention. Then the coolant temperature is lowered to cover the remaining set points to obtain heat fluxes and heat transfer coefficients at different subcoolings.

Heat transfer coefficient is defined as described in the main text. Its computation, and the uncertainty propagation procedure can be seen in our previous work.[1]

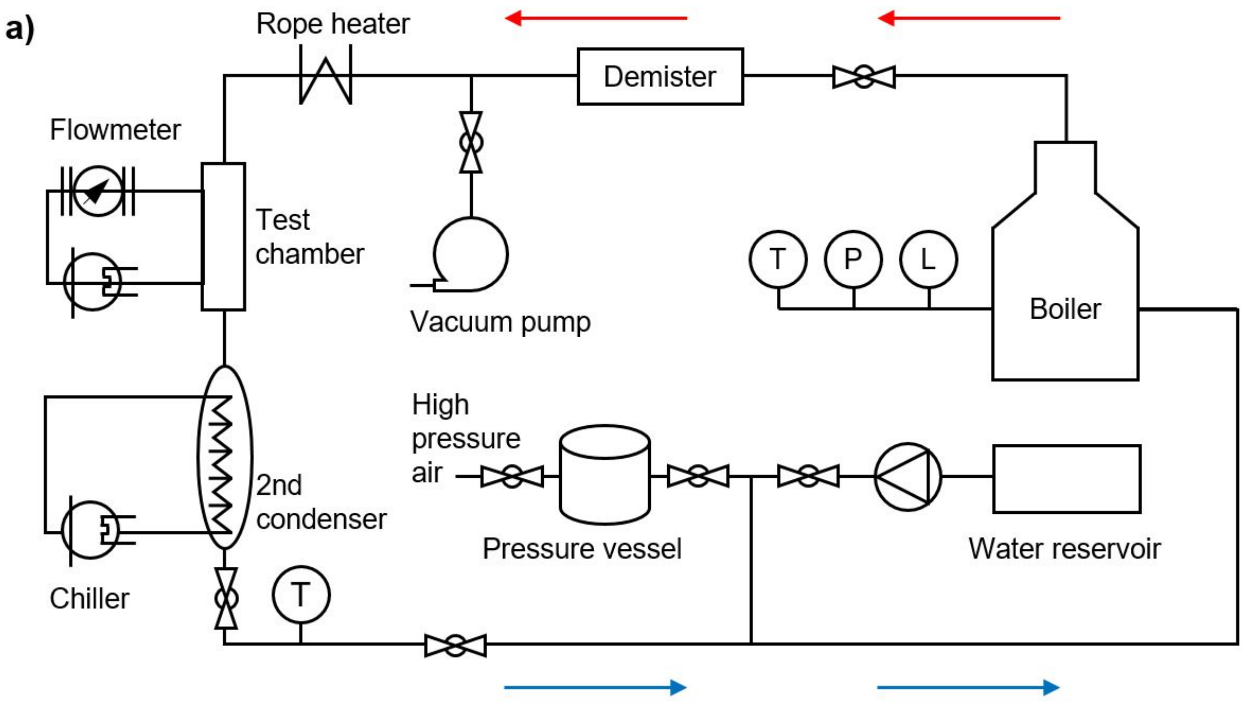

Figure S7. Device for condensation heat transfer measurements at 1.4 bar. a) Schematic of the overall experimental setup. For high-pressure heat transfer measurement, we follow similar procedures to our previous work,[6] which is referred to for details. The high-pressure flow chamber is installed into a loop so that experiments can be run continuously for an extended period. To fully degas the water and exclude the non-condensable gases, the condensation chamber is initially vacuumed to below 20 mbar. After that, the whole setup it is filled with liquid water by opening the connection with a water reservoir. To further reduce the amount of non-condensable gases, water is repeatedly pumped and a valve at the highest point of the setup is repeatedly opened to release the air residues. During this process the pressure in the setup is always kept above the atmospheric pressure. For surface temperature, a thermocouple is installed into the substrate. During experiments, the steam pressure is kept at 1.42 bar and the mean steam speed in the channel is controlled at 3 m·s^{-1} or 9 m·s^{-1}.

b)

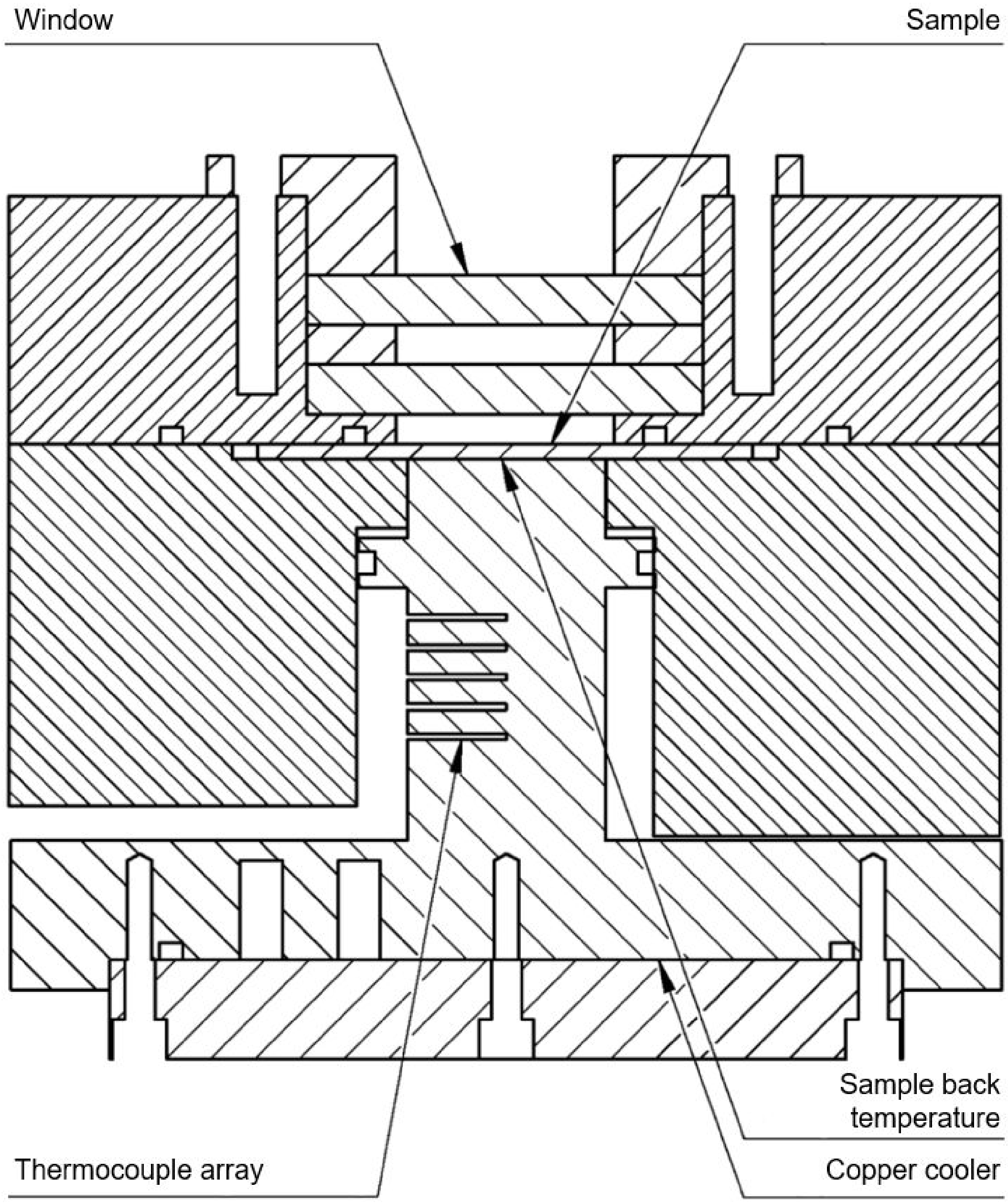

Figure S7. Device for condensation heat transfer measurements at 1.4 bar. b) Schematic of the condensation chamber, top-view cross-section. Heat transfer coefficient is defined as described in the main text. Its computation, and the uncertainty propagation procedure can be seen in our previous work.[6]

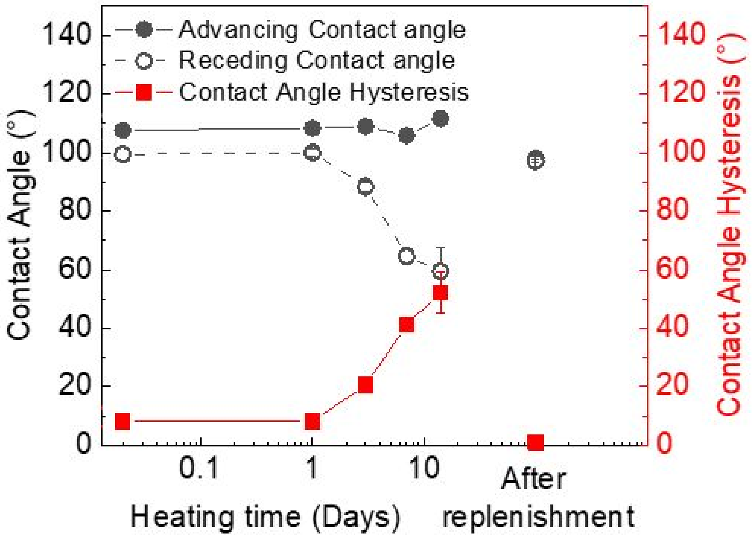

Figure S8. Durability test of PDMS-coated copper in hot water (~100 °C) and its recovery. After several days in hot water, the surface lost its wetting property towards water with a high contact angle hysteresis. After replenishment by applying a bit of PDMS oil on top, low contact angle hysteresis is achieved again.

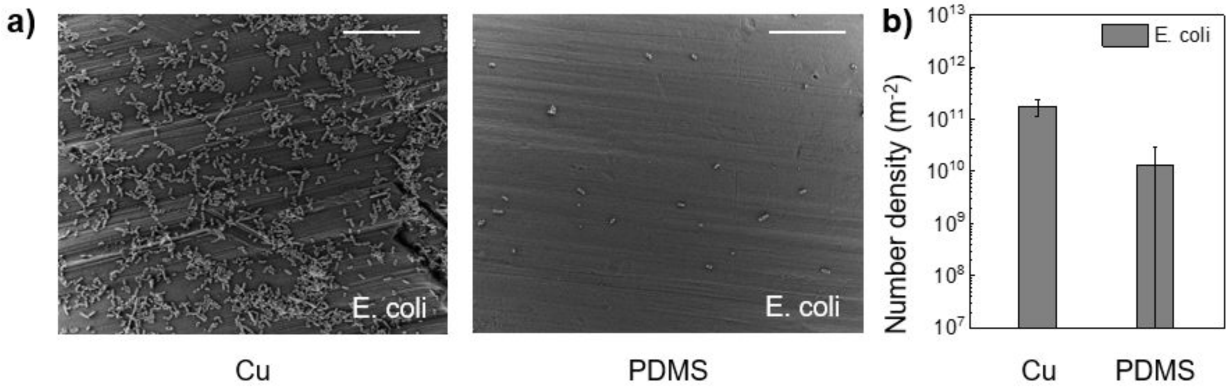

Figure S9. Antifouling property of different surfaces. a) SEM images of E. coli on pristine copper and PDMS-coated copper. Scale bar: 20 μm. b) Number density of attached bacteria on the two surfaces.

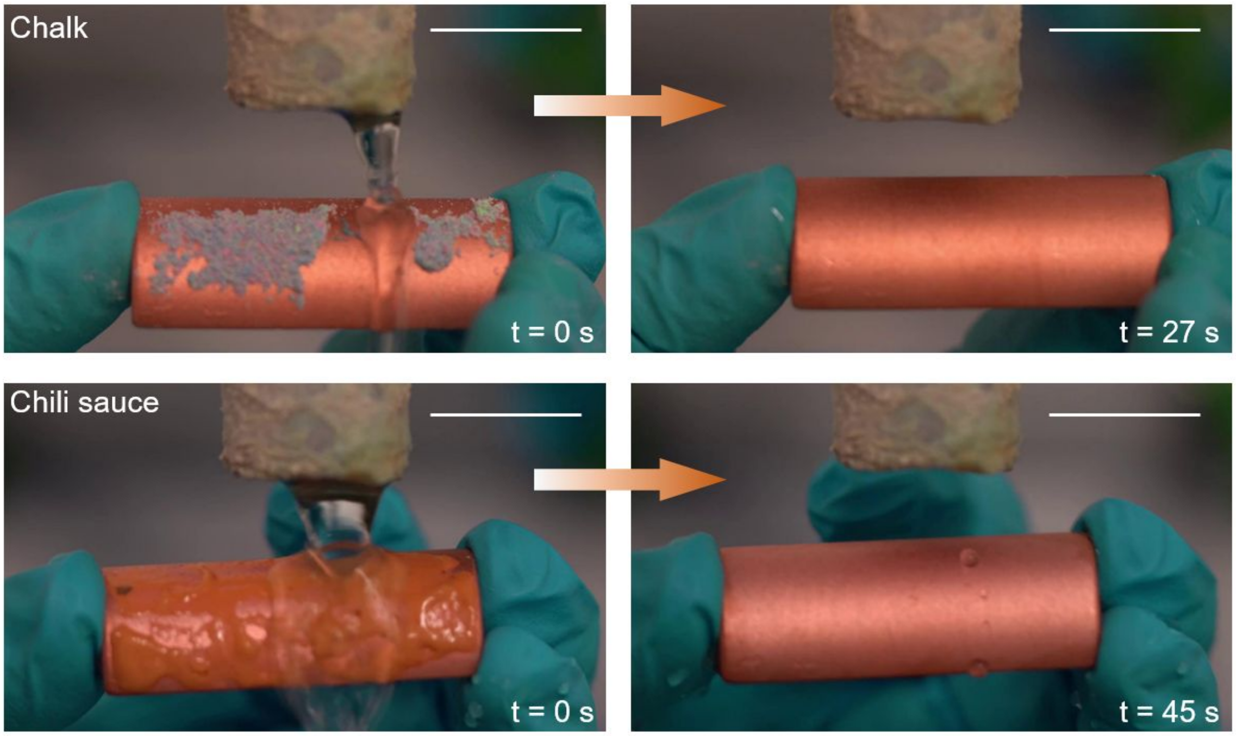

Figure S10. Self-cleaning effect. Photographs showing the self-cleaning property of PDMS brushes after being contaminated by chalk powder and chili sauce, when rinsed with tap water. Scale bar: 20 mm.

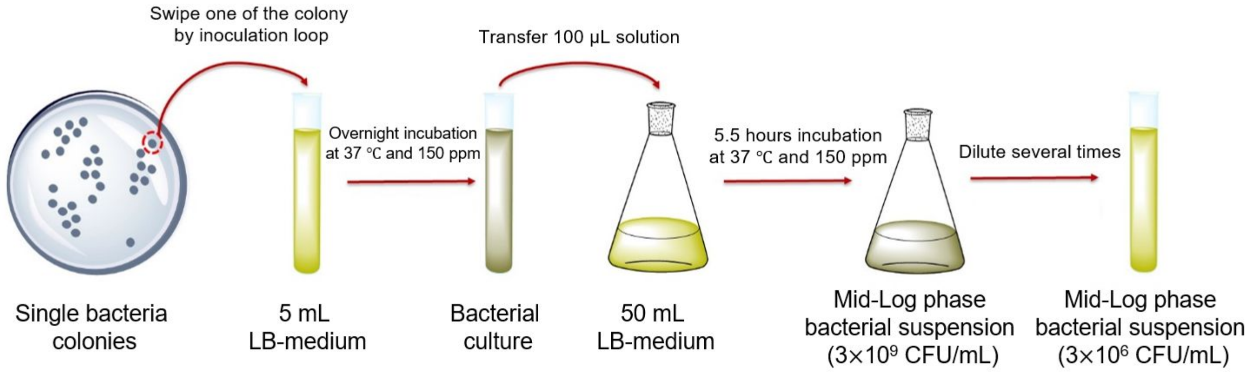

Figure S11. Bacterial suspension preparation. Schematic shows the experimental procedure of bacteria culture and solution preparation.

Bacteria adhesion of gram-negative (*Escherichia coli* (*E. coli*), MG1655) and gram positive (*Staphylococcus aureus* (*S. aureus*), DSM 11823) bacteria on the coating surfaces are examined by scanning electron microscopy (SEM, LEO 1530 Gemini, Zeiss). Mid-Log phase bacterial suspension for the test is prepared by the following procedure: a single bacteria colony inoculate into a test tube containing 5 mL LB (Luria-Bertani) medium (2.5% LB (Invitrogen) in Milli-Q water). Then the tube is shaken in a half-covered position in an orbital shaker incubator (Eppendorf™ Innova™ 44) at 37°C and 150 rpm for overnight. Then, 100 µL the overnight bacteria suspension is transferred into 50 mL fresh LB-medium and incubated under stirring, using an incubator at 37°C and 200 rpm for 5.5 hours to reach the mid-Log phase which contained $3x10^9$ colony forming units for per milliliter (CFU/mL). 1 mL mid-Log phase inoculum is diluted several times (10^1, 10^2 and 10^3) with fresh LB-medium to gain $3x10^6$ CFU/mL to prepare bacteria test suspension.

Tables

Table S1. Thickness of the current state-of-the-art superhydrophobic surfaces.

Author/Journal	Composition	Thickness (μm)
Miljkovic, Nenad, et al. *Nano Letters* 13.1 (2013): 179-187.	CuO + tridecafluoro-(1,1,2,2-tetrahydrooctyl)-1-trichlorosilane	1
Tsoi, Shufen, et al. *Langmuir* 20.24 (2004): 10771-10774.	SiO_2 + 3,3,3-trifluoropropylsiloxane	2
Tang, Yu, et al. *Nano Letters* 21.22 (2021): 9824-9833.	Copper + alkaline oxidation	>8
Wen, Rongfu, et al. *Joule* 2.2 (2018): 269-279.	Copper Nanowire + trichloro (1H,1H,2H,2H-perfluorooctyl)-silane	>15
Wang, Dehui, et al. *Nature* 582.7810 (2020): 55-59.	Armor microstructures + silica nanomaterial	>25
Wu, Shuwang, et al. *Proceedings of the National Academy of Sciences* 117.21 (2020): 11240-11246.	soot particles + tetraethoxysilane + polydimethylsiloxane	1.5-30
Zhang, Jilin, et al. *Macromolecular Rapid Communications* 25.11 (2004): 1105-1108.	PTFE	50
Varanasi, Kripa K., et al. *Applied Physics Letters* 97.23 (2010): 234102.	silicon posts + tridecafluoro-1,1,2,2-tetrahydrooctyl trichlorosilane	>100
Peng, Chaoyi, et al. *Nature Materials* 17.4 (2018): 355-360.	fluorinated epoxy + perfluoropolyether	>150
Liu, Yahua, et al. *Nature Physics* 10.7 (2014): 515-519.	Copper + tapered posts + trichloro(1H,1H,2H,2H-perfluorooctyl)silane	400

Table S2. Thickness of the current state-of-the-art lubricant infused surfaces.

Author/Journal	Composition	Thickness (μm)
Tripathy, Abinash, et al. *ACS Nano* 15.9 (2021): 14305-14315.	Vertical Graphene + Krytox 1525	0.07
Preston, Daniel J. et al. *Scientific Reports* 8.1 (2018): 540.	CuO + Krytox GPL 101	1
Sett, Soumyadip, et al. *Nano Letters* 19.8 (2019): 5287-5296.	CuO + Krytox 1525	>1
Sun, Jianxing, et al. *Soft Matter* 15.24 (2019): 4808-4817.	SiO_2 + Krytox GPL 102	>1.5
Li, Shuai, et al. *Langmuir* 38.41 (2022): 12610-12616.	PDMS	>2
Tenjimbayashi, Mizuki, et al. *Langmuir* 34.4 (2018): 1386-1393.	Poly(vinylidene fluoride-co-hexafluoropropylene) + Dibutyl phthalate +Perfluoropolyether	4.24
Anand, Sushant, et al. *ACS Nano* 6.11 (2012): 10122-10129.	Silicon micro-post + Krytox	10
Wooh, Sanghyuk, et al. *Angewandte Chemie* 129.18 (2017): 5047-5051.	TiO_2 + PDMS	>10
Ge, Qiaoyu, et al. *ACS Applied Materials & Interfaces* 12.19 (2020): 22246-22255.	Etched Aluminum + Krytox 1506	20-50
Wong, Tak-Sing, et al. *Nature* 477.7365 (2011): 443-447.	Teflon + perfluoropolyether	60-80

Table S3. Cost estimation of PDMS brushes on copper plate. The total cost for PDMS brushes coating is 9.43 USD per m^2. The price for the chemicals and electricity can be found from reference.[7-9]

Item	Price ($)	Amount ($\cdot 1m^{-2}$ copper)	Cost ($)
Poly(dimethylsiloxane)	2.22 /kg	0.97 g	2.15
Electricity	0.14 /kWh		
Plasma 120W		5min, 0.2 kWh	0.28
Oven 1250W (max)		24h, 1.5 kWh	0.21
Acetone	0.86 /kg	7.89kg	6.79
Total			9.43

Supporting Videos

Supporting Video S

Supporting Video S1: Water drop slide off a PDMS-coated copper tube. Tilt angle: 25°.

Supporting Video S2: Water drop sliding on PDMS brushes coated copper plate: 1st drop.

Supporting Video S3: Water drop sliding on PDMS brushes coated copper plate: 100th drop.

Supporting Video S4: Water drop sliding on PDMS brushes coated copper plate: 1000th drop.

Supporting Video S5: Water drop sliding on PDMS brushes coated copper plate: 5000th drop.

Supporting Video S6: Left: Steam condensation on a hydrophilic copper oxide surface. Right: real-time heat transfer coefficient and subcooling. Steam pressure: 30 mbar. Playback: 240×.

Supporting Video S7: Left: Steam condensation on a superhydrophobic aluminium surface. Right: real-time heat transfer coefficient and subcooling. Steam pressure: 30 mbar. Playback: 240×. The video is adapted from our previous work.[10]

Supporting Video S8: Left: Steam condensation on PDMS brushes coated copper surface. Right: real-time heat transfer coefficient and subcooling. Steam pressure: 30 mbar. Playback: 240×.

Supporting Video S9: Steam condensation on PDMS brushes coated copper surface. Steam pressure: 1.4 bar. Playback: 0.1×.

Supporting Video S10: Cleaning process of PDMS brushes coated copper plate contaminated with chalk powder.

Supporting Video S11: Cleaning process of PDMS brushes coated copper plate being contaminated with chili sauce.

Present Addresses:

#C.W.E.L.: Massachusetts Institute of Technology, Cambridge, MA, 02139, United States

Supporting References

(1) Tripathy, A.; Lam, C. W. E.; Davila, D.; Donati, M.; Milionis, A.; Sharma, C. S.; Poulikakos, D. Ultrathin Lubricant-Infused Vertical Graphene Nanoscaffolds for High-Performance Dropwise Condensation. *ACS Nano* **2021**, *15* (9), 14305-14315. DOI: 10.1021/acsnano.1c02932.

(2) Khalil, K.; Soto, D.; Farnham, T.; Paxson, A.; Katmis, A. U.; Gleason, K.; Varanasi, K. K. Grafted Nanofilms Promote Dropwise Condensation of Low-Surface-Tension Fluids for High-Performance Heat Exchangers. *Joule* **2019**, *3* (5), 1377-1388. DOI: 10.1016/j.joule.2019.04.009.

(3) Hou, Y.; Yu, M.; Shang, Y.; Zhou, P.; Song, R.; Xu, X.; Chen, X.; Wang, Z.; Yao, S. Suppressing Ice Nucleation of Supercooled Condensate with Biphilic Topography. *Phys. Rev. Lett.* **2018**, *120* (7), 075902. DOI: 10.1103/PhysRevLett.120.075902.

(4) Ouellette, R. J.; Rawn, J. D. *Organic Chemistry Study Guide: Key Concepts, Problems, and Solutions*; Elsevier, **2014**.

(5) John, A. D. Lange's handbook of chemistry. In *Universitas Of Tennese Knoxville, Fifthenth Edition, Mc. Graw Hill Inc, New York. Conference*, **1999**.

(6) Donati, M.; Lam, C. W. E.; Milionis, A.; Sharma, C. S.; Tripathy, A.; Zendeli, A.; Poulikakos, D. Sprayable Thin and Robust Carbon Nanofiber Composite Coating for Extreme Jumping Dropwise Condensation Performance. *Adv. Mater. Interfaces* **2020**, *8* (1), 2001176. DOI: 10.1002/admi.202001176.

(7) Company, T. C. *Polydimethylsiloxane (PDMS), Packaging Size: 200 Kg, Packaging Type: Barrel.* **2022**. https://www.indiamart.com/proddetail/polydimethylsiloxane-pdms-14734166255.html.

(8) *Acetone prices*. **2022**. https://www.chemanalyst.com/Pricing-data/acetone-12.

(9) *Electricity prices*. **2022**. https://www.globalpetrolprices.com/electricity_prices.

(10) Sarkiris, P.; Constantoudis, V.; Ellinas, K.; Lam, C. W. E.; Milionis, A.; Anagnostopoulos, J.; Poulikakos, D. a.; Gogolides, E. Topography Optimization for Sustainable Dropwise Condensation: The Critical Role of Correlation Length. *Adv. Funct. Mater*. **2023**, 2306756. DOI: 10.1002/adfm.202306756.
